# Noise Provides New Insights on Contrast Sensitivity Function

**DOI:** 10.1371/journal.pone.0090579

**Published:** 2014-03-13

**Authors:** Ge Chen, Fang Hou, Fang-Fang Yan, Pan Zhang, Jie Xi, Yifeng Zhou, Zhong-Lin Lu, Chang-Bing Huang

**Affiliations:** 1 Key Laboratory of Behavioral Science, Institute of Psychology, Chinese Academy of Sciences, Beijing, China; 2 University of Chinese Academy of Sciences, Beijing, China; 3 Center for Cognitive and Brain Sciences, Department of Psychology, Ohio State University, Columbus, Ohio, United States of America; 4 Key Laboratory of Brain Function and Diseases, Chinese Academy of Sciences, and School of Life Sciences, University of Science and Technology of China, Hefei, Anhui, China; University of Salamanca- Institute for Neuroscience of Castille and Leon and Medical School, Spain

## Abstract

Sensitivity to luminance difference, or contrast sensitivity, is critical for animals to survive in and interact with the external world. The contrast sensitivity function (CSF), which measures visual sensitivity to spatial patterns over a wide range of spatial frequencies, provides a comprehensive characterization of the visual system. Despite its popularity and significance in both basic research and clinical practice, it hasn’t been clear what determines the CSF and how the factors underlying the CSF change in different conditions. In the current study, we applied the external noise method and perceptual template model to a wide range of external noise and spatial frequency (SF) conditions, and evaluated how the various sources of observer inefficiency changed with SF and determined the limiting factors underlying the CSF. We found that only internal additive noise and template gain changed significantly with SF, while the transducer non-linearity and coefficient for multiplicative noise were constant. The 12-parameter model provided a very good account of all the data in the 200 tested conditions (86.5%, 86.2%, 89.5%, and 96.4% for the four subjects, respectively). Our results suggest a re-consideration of the popular spatial vision model that employs the CSF as the front-end filter and constant internal additive noise across spatial frequencies. The study will also be of interest to scientists and clinicians engaged in characterizing spatial vision deficits and/or developing rehabilitation methods to restore spatial vision in clinical populations.

## Introduction

Sensitivity to luminance difference, or contrast sensitivity, is critical for animals to survive in and interact with the external world, e.g. searching for food, locating water source, mating, socializing, and disclosing predators from its surrounding camouflages [1]. The CSF, which measures visual sensitivity to spatial patterns over a wide range of spatial frequencies, provides a comprehensive characterization of the visual system [2]–[6]. A typical CSF resembles a band-pass filter that peaks at intermediate frequencies (usually 2–6 c/deg) and drops off at both lower and higher spatial frequencies [7]–[12]. The CSF has been found to differ greatly among species [13], improve with development [14], deteriorate with aging [15], and vary with attentional state [16], [17], luminance level [18], adaptation [19], and a variety of visual diseases, including amblyopia [20]–[22], glaucoma [23], [24], dyslexia [25], and major depressive disorder [26], [27]. Moreover, the pattern of CSF changes may vary with clinical conditions. For example, amblyopia is largely regarded as a high SF deficit [28], [29] and dyslexia as a condition with low SF deficits ([30], [31], but see [32]).

Models of spatial vision that employ the CSF as the front-end SF filter have been developed to account for human performance in a wide range of visual tasks, including letter identification [33], [34] and face recognition [35], implicitly treating the CSF as the gain profile of the visual system in the spatial frequency space [36]. Although these models provided good accounts of human performance in many tasks, equating the gain profile of the channels according to the CSF of the observer may be problematic. With a contrast matching paradigm, Georgeson and Sullivan [36] have shown that the perceived contrast of gratings is largely independent of the testing spatial frequencies in high contrast conditions, a finding indicative of almost equal gain across different spatial frequencies in the visual system. Several other studies have also found that adding high magnitude of external noise to the to-be-detected gratings can flatten the CSF, suggesting that the CSF is not a simple function of the gain of the visual system [20], [37]–[40]. In addition, there are also many known nonlinearities, including nonlinear transducer function and multiplicative noise in the visual system [41]–[43], which will inevitably perplex the interpretation of previous data about CSF. The spatial vision models that consist of CSF as the front-end filter and constant internal additive noise across spatial frequencies may account for human performance in certain conditions but not in more extended conditions that include a wide range of stimulus contrasts, external noise, and performance levels [44]. Those models need to be elaborated by fully specifying the gain, non-linearity, and noises in the visual system [45]. In the present paper, we attempt to directly determine the perceptual limitations underlying the contrast sensitivity functions by combining the external noise method and observer modeling. The result will allow us to test the traditional multi-channel model of spatial vision and provide new insights for understanding CSF deficits in clinical conditions.

Applications of the external noise method and observer models to identify the intrinsic limitations of human observers date back to the 1950’s [46]. The external noise method measures the threshold versus external noise contrast (TvC) function to estimate how much signal contrast or feature difference is required for an observer to maintain a certain performance level as a function of external noise [41], [43], [46], [47]. The TvC function is usually fitted with a specific observer model such as the linear amplifier model (LAM) [48], the induced noise model [49], the linear amplifier model with decision uncertainty [43], the induced noise and uncertainty model [42], or the perceptual template model [41]. The perceptual template model (PTM) incorporates and integrates the major components of the previous observer models and has been shown to provide an excellent account of a range of psychophysical data [45].

To date, the external noise paradigm and observer models have been usually implemented in a single SF condition. In the current study, we apply the external noise method to characterize the limiting factors underlying contrast sensitivity at different spatial frequencies [41], [45]. The PTM is used to decompose contrast sensitivity into four intrinsic limitations of the perceptual system [45]: (1) the gain of the perceptual template, (2) a nonlinear transducer, (3) internal multiplicative noise whose amplitude is directly related to the total amount of input stimulus energy, and (4) internal additive noise whose amplitude is invariant with input stimulus. Characterizing the four factors as functions of SF allows us to identify the perceptual limitations underlying the CSF.

## Methods

### Subjects

Four novice adult observers, two males and two females, aged 21–27 years (24.25±2.50; mean ± s.d.), with normal or corrected-to-normal vision, participated in the study. All studies were performed with written informed consent of the subjects and approved by the research ethics committee of Institute of Psychology, CAS and followed the tenets of the Declaration of Helsinki.

### Apparatus and Stimuli

All stimuli were generated by a PC running Matlab and PsychToolBox extensions [50], [51] and presented on a luminance-linearized Sony G520 monitor [52] with a vertical refresh rate of 85Hz, a resolution of 1600×1200 pixels and mean luminance of 30.6 cd/m^2^. A special circuit was used to combine two 8-bit output channels of the video card to produce 14 bits of gray levels [52].

**Figure 1:**
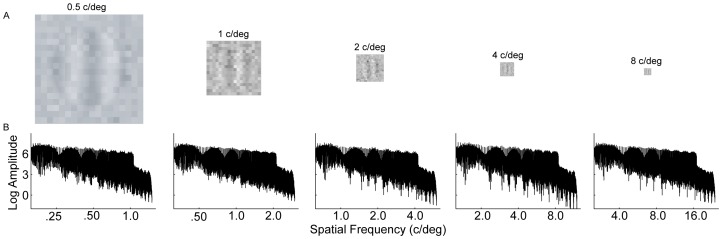
Stimuli samples and the spectra of external noise. (A) Noise masked gratings in different spatial frequency conditions (*N_ext_* = 0.32). (B) The magnitude spectra of external noise in different spatial frequency conditions. Note that different ranges are used for the x-axis in plotting the magnitude spectra in different spatial frequency conditions.

Signal stimuli were vertical sinusoidal gratings at five spatial frequencies (SF: 0.5, 1, 2, 4, and 8 c/deg), presented binocularly in the center of the display. Each grating consisted of three cycles. The size of the gratings was inversely proportional to their spatial frequencies (i.e., 576, 288, 144, 72, and 36 pixels), subtending 6, 3, 1.5, 0.75, and 0.375 degrees of visual angle at a viewing distance of 138.5 cm. External noise images, constructed from Gaussian distributed pixel intensities with eight standard deviations (0, 0.01, 0.02, 0.04, 0.08, 0.16, 0.24, and 0.32), were combined with signal gratings through temporal integration (**Figure 1** **. A**). The size of the signal grating and external noise images was the same. The size of the noise elements was scaled with the signal grating size to maintain 18 noise elements per image so that the spectra of signal gratings and the external noise maintained a constant relationship across different spatial frequency conditions (**Figure 1**).

### Design

In the main experiment, we adopted the method of constant stimuli (MCS) to obtain psychometric functions (PFs) in all forty conditions (5 SFs×8 external noise levels). Each PF was sampled at five signal contrasts with 90 trials per sample. There were 200 conditions (5 SFs×8 external noise levels×5 signal contrasts), and a total of 18,000 trials per observer.

Each observer ran 15 sessions of 1200 trials each. A mini-block design was used: the 1200 trials were divided into 6 blocks, each of which consisted of five mini-blocks of 40 trials (8 external noise levels×5 signal contrast×5 repetitions) in a single SF condition. All five SF conditions were tested in each block. External noise and signal contrast conditions in each mini-block were randomized. The order of SF was randomized in each block. Observers could volunteer for a break after every 40 trials. A mandatory one-minute break was provided every 80 trials. Each session took approximately 75 minutes.

To ensure efficient sampling of the PFs, a Bayesian procedure was first utilized to quickly estimate contrast thresholds at 70.7% and 79.4% correct in detecting gratings of the five spatial frequencies in the zero and highest external noise conditions in a pilot study [61]. Rough estimates of the contrast thresholds in the remaining noise conditions were linearly interpolated from these thresholds. For a given SF and external noise level, we sampled the PF at five signal contrast levels using an efficient sampling method [53].

### Procedure

**Figure 2:**
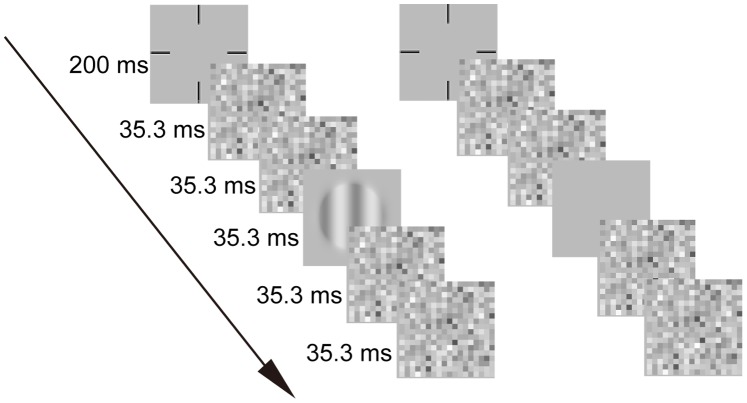
The procedure of one trial. Each trial consisted of two intervals separated by 505.9

Subjects performed a two-interval forced-choice (2-IFC) grating detection task in both the main and pilot experiments. **Figure 2** depicts a typical trial. Each trial started with a 200-ms fixation cross in the center of the display, followed by two stimulus presentation intervals, each demarcated by a brief tone and consisted of a sequence of 35.3-ms frames: two external noise frames, one signal or blank frame, and two additional external noise frames. The two intervals were separated by a 505.9-ms inter-stimulus interval. Signal appeared in one of the two intervals with equal probability. Subjects were asked to report which interval contained the signal by a keypress. All external noise frames were sampled independently. No feedback was given.

## Results

### Psychometric Functions

**Figure 3:**
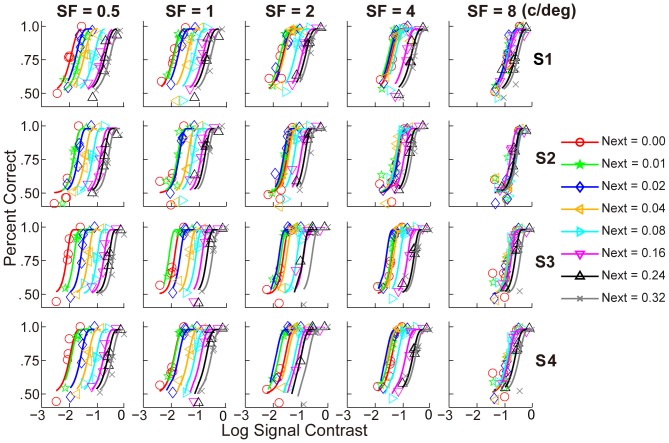
Contrast psychometric functions for the four observers. Each row contains contrast psychometric functions for one observer, with increasing spatial frequency from left to right columns. Different colors indicate different external noise conditions. The solid line represents the best-fitting model in which the contrast psychometric functions shared the same slope across different spatial frequencies and external noise levels. See details in Table 1.

**Figure 3** shows the PFs in all forty conditions. For each observer, the PFs shifted to the right as external noise increased in low to medium spatial frequencies, and tended to collapse in high spatial frequencies. The pattern of results suggested that contrast threshold increased with external noise, and the rate of increase was slower in high spatial frequencies.

We evaluated the relationship between the slopes and thresholds of the forty PFs for each observer through model comparison. Each PF was fitted with a Weibull function:
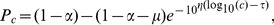
(1)where *P_c_* is percent correct in each condition, *α* is the lapsing rate and set to be 0.02, *μ* is the guessing rate of 0.5, *η* is the slope of the PF, and *τ* is the contrast threshold at 80.3% performance level. We tested four different models: (1) the full model: all parameters are different for the 40 PFs, leading to a total of 80 parameters (40 slopes and 40 thresholds); (2) reduced model 1: the slopes are the same but the thresholds are different across the PFs; (3) reduced model 2: the thresholds are the same but the slopes are different across the PFs; (4) the most reduced model: the slopes and thresholds are the same across the PFs. The four models were fitted to the PFs with a maximum likelihood procedure, gauged by an *r* ^2^ statistics, and compared with Chi-square test for nested models (Eq. 2–4).



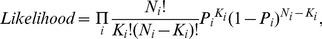
(2)

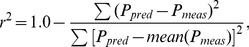
(3)




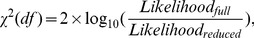
(4)where *i* denotes a single condition, *N*_i_ and *K*_i_ are the numbers of the total and correct trials, *P_i_* is the percent correct derived from the Weibull function; *P*_pred_ and *P*_meas_ are the predicted and measured percent correct, respectively; *Likelihood_full_* and *Likelihood_reduced_* are the maximum likelihood of the full and reduced models, respectively, *df* is equal to the difference of the number of parameters of the models.

The model with the same slope but different thresholds across all spatial frequencies and external noise levels accounted for 89.6%, 90.2%, 91.9%, and 95.8% of the variance for the four observers, respectively. Model comparison revealed that this model was statistically comparable to the full model (both slope and threshold vary; see **Table 1**) and superior to the most reduced model (neither slope nor threshold varies; see **Table 1**). The slope estimated from the model was 2.15, 3.12, 2.83, and 3.02 (2.78±0.44) for the four observers, respectively (see **Table 2** **)**. The values were comparable to estimates reported in the literature [43], [48], [54].

**Table 1:** Chi-square test for nested models which were estimated from Weibull functions.

	*df*	S1		S2		S3		S4	
		χ^2^	*p*	χ^2^	*p*	χ^2^	*p*	χ^2^	*p*
M1 vs. M2	39	18.76	1.00	15.53	1.00	18.76	1.00	15.53	1.00
M1 vs. M3	39	954.32	0.00	1047.54	0.00	954.32	0.00	1047.54	0.00
M1 vs. M4	78	973.84	0.00	1051.69	0.00	973.84	0.00	1051.69	0.00
M2 vs. M4	39	955.08	0.00	1036.16	0.00	955.08	0.00	1036.16	0.00
M3 vs. M4	39	19.52	1.00	4.15	1.00	19.52	1.00	4.15	1.00

M1 is the full model with both slope and threshold varying across the 40 PFs; M2 is the reduced model that has the same slope across the 40 PFs; M3 is another reduced model with the same threshold across the 40 PFs; M4 is the most reduced model that has the same slope and threshold in all 40 PFs; *df* is the degree of freedom. For all four observers, M2 is the only model that is comparable to M1 (the full model) and superior to M4 (the most reduced model).

**Table 2:** Contrast thresholds estimated from the best-fitting Weibull function.

Subject	SF(c/deg)	Log10(Contrast threshold)							
		Noise level (*N_ext_*)							
		0.00	0.01	0.02	0.04	0.08	0.16	0.24	0.32
S1	0.5	−1.92	−1.59	−1.74	−1.44	−1.09	−0.78	−0.70	−0.52
	1	−1.90	−1.88	−1.73	−1.42	−1.15	−0.85	−0.72	−0.56
	2	−1.63	−1.72	−1.60	−1.58	−1.20	−0.96	−0.83	−0.72
	4	−1.39	−1.51	−1.46	−1.34	−1.26	−0.91	−0.69	−0.56
	8	−0.84	−0.90	−0.93	−0.85	−0.86	−0.84	−0.60	−0.50
S2	0.5	−1.81	−1.81	−1.66	−1.42	−1.15	−0.86	−0.74	−0.58
	1	−1.73	−1.76	−1.70	−1.39	−1.21	−0.91	−0.79	−0.67
	2	−1.49	−1.55	−1.61	−1.43	−1.23	−1.00	−0.86	−0.72
	4	−1.14	−1.25	−1.17	−1.25	−1.22	−0.94	−0.78	−0.67
	8	−0.64	−0.77	−0.61	−0.57	−0.55	−0.77	−0.64	−0.56
S3	0.5	−2.06	−1.83	−1.57	−1.29	−0.90	−0.76	−0.57	−0.41
	1	−1.89	−1.93	−1.73	−1.33	−1.12	−0.89	−0.71	−0.53
	2	−1.50	−1.75	−1.73	−1.52	−1.18	−1.02	−0.93	−0.63
	4	−1.37	−1.44	−1.43	−1.41	−1.15	−0.85	−0.60	−0.50
	8	−0.79	−0.76	−0.85	−0.86	−0.86	−0.76	−0.58	−0.44
S4	0.5	−2.14	−1.88	−1.77	−1.51	−1.21	−0.95	−0.81	−0.68
	1	−2.00	−1.89	−1.84	−1.69	−1.28	−1.01	−0.92	−0.77
	2	−1.77	−1.74	−1.76	−1.59	−1.24	−1.04	−0.89	−0.74
	4	−1.40	−1.43	−1.48	−1.42	−1.22	−0.95	−0.77	−0.67
	8	−0.88	−0.87	−0.88	−0.90	−0.88	−0.76	−0.74	−0.62

### Contrast Sensitivity Functions

**Figure 4:**
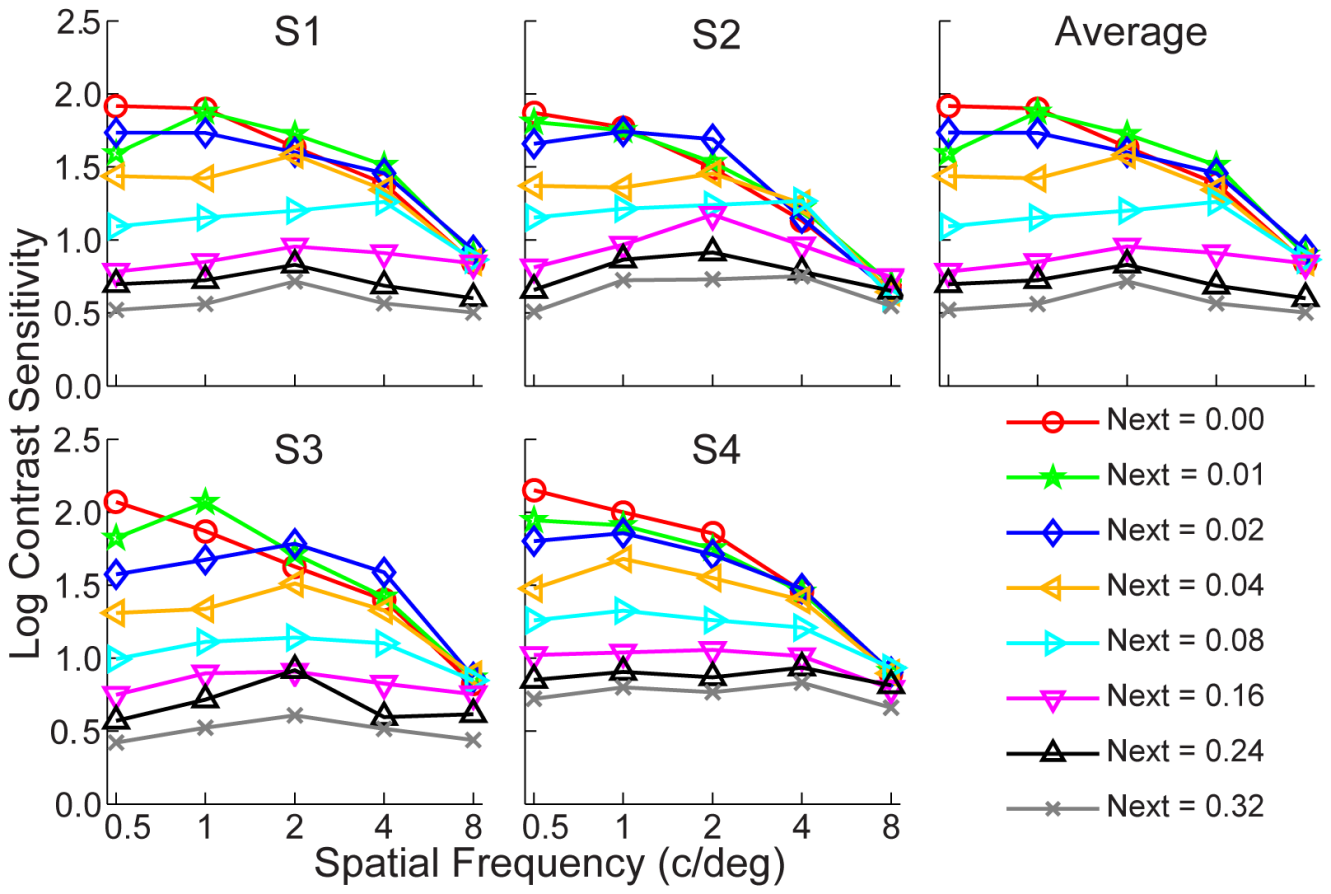
Contrast sensitivity functions in different external noise conditions. Different colors indicate different external noise conditions.

CSFs corresponding to 80.3% correct performance that were derived from the best fitting results are plotted in **Figure 4** in eight external noise conditions for each observer (also see **Table 2**). In zero external noise, the CSFs showed a typical band-pass or low-pass profile. As external noise increased, contrast sensitivity decreased, and the CSFs became increasingly flat. Averaged over observers and spatial frequencies, contrast sensitivity decreased from 47.27 to 3.80, a reduction of 91.8%, as external noise increased from 0 to 0.32. Contrast sensitivity varied significantly with SF (*F* (4, 12) = 169.11, *p* = 1.93×10^−10^), external noise levels (*F* (7, 21) = 163.02, *p* = 7.54×10^−17^), and their interaction (*F* (28, 84) = 27.14, *p* = 3.16×10^−10^).

### PTM Model Analysis

The perceptual template model described performance accuracy (*d*’) as a function of signal and external noise contrasts:
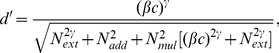
(5)where *c* is the signal contrast, *β* is the gain of the perceptual template(relative to its gain to external noise), *γ* characterizes system’s non-linearity, *N_add_* is the standard deviation of the internal additive noise, *N_ext_* is the standard deviation of external noise, *N_mul_* is the proportional constant for multiplicative noise. In the PTM, both signal and external noise are processed through the perceptual system; perceptual decisions are made based on the inputs to the decision unit, without knowing what signal and what noise is. Eq. 5 describes the input-output relationship in the PTM. It does not imply that signal and external noise are known independently by the PTM. Performance accuracy (*d’*) can also be expressed as:
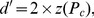
(6)where z(Pc) is the z-score of the 2-IFC percent correct. Combing Eq.5 and 6, we can fit the PTM to the measured PFs and evaluate how the PTM parameters vary across spatial frequencies (**Table 3**). We also constructed a model lattice ranging from a model with a single set of parameters across all SF conditions to a model with every single parameter varying with SF.

**Table 3:** Parameters for the best fitting perceptual template model (PTM).

	SF(c/deg)	0.5	1	2	4	8
S1	*N_add_*	7.64×10^−6^	4.25×10^−6^	3.94×10^−5^	1.24×10^−4^	2.10 ×10^−3^
	*β*	1.40	1.61	2.02	1.62	1.26
	*N_mul_*	0.28				
	*γ*	3.01				
S2	*N_add_*	4.46×10^−6^	1.24×10^−5^	1.09×10^−4^	6.72×10^−4^	1.14×10^−2^
	*β*	1.39	1.63	2.20	1.85	1.49
	*N_mul_*	0.25				
	*γ*	3.04				
S3	*N_add_*	7.50×10^−7^	6.44×10^−6^	3.63×10^−5^	1.09×10^−4^	3.41×10^−3^
	*β*	0.97	1.38	1.66	1.32	1.21
	*N_mul_*	0.22				
	*γ*	2.84				
S4	*N_add_*	8.46×10^−6^	3.47×10^−5^	8.62×10^−5^	5.71×10^−4^	1.06×10^−2^
	*β*	1.81	2.29	2.03	1.88	1.67
	*N_mul_*	0.21				
	*γ*	2.59				

The best fitted model which allowed both *N_add_* and *β* to vary with SF provided comparable accounts (*r^2^* = 86.5%, 86.2%, 89.5%, and 96.4% for the four observers, respectively) of the variance of the data with the full model that allowed all four parameters free to vary, and was superior to all its reduced versions, i. e., the model with only *N_add_* free to vary with SF, the model with only *β* free to vary with SF, and the model with the same parameters across SFs (see details in **Table 4**). In other words, the CSFs measured in a wide range of external noise conditions could be explained by SF-dependent internal noise *N_add_* and template gain *β*, and constant non-linear transducer and multiplicative noise coefficient.

**Table 4:** Results of PTM model comparisons.

		S1		S2		S3		S4	
	*df*	χ^2^	p	χ^2^	p	χ^2^	p	χ^2^	p
M5 vs. M1	16	438	0.00	898	0.00	832	0.00	622	0.00
M5 vs. M2	12	41	4.92×10^−5^	53	4.21×10^−7^	149	0.00	29	1.60×10^−3^
M5 vs. M3	12	342	0.00	826	0.00	490	0.00	416	0.00
M5 vs. M4	8	2	0.97	3	0.93	2	0.99	3	0.91
M4 vs. M1	8	435	0.00	895	0.00	831	0.00	619	1.15×10^−5^
M4 vs. M2	4	39	8.27×10^−8^	50	3.83×10^−8^	147	0.00	26	0.00
M4 vs. M3	4	340	0.00	823	0.00	489	0.00	413	0.00
M3 vs. M1	4	96	0.00	72	8.88×10^−15^	342	0.00	206	0.00
M2 vs. M1	4	397	0.00	845	0.00	684	0.00	593	0.00

M1: the most reduced model with all four PTM parameters constant across SFs; M2: reduced model with only internal additive noise varying across SFs; M3: reduced model with changing template gain over SFs; M4: another reduced model with both *N_add_* and *β* varying across SFs; M5: the full model with *N_add_*, *β*, *N_mul_*, and *γ* changing with SFs. The Chi-square test for nested model analysis shows that the fourth model (M4) is the best fitting model.

**Figure 5:**
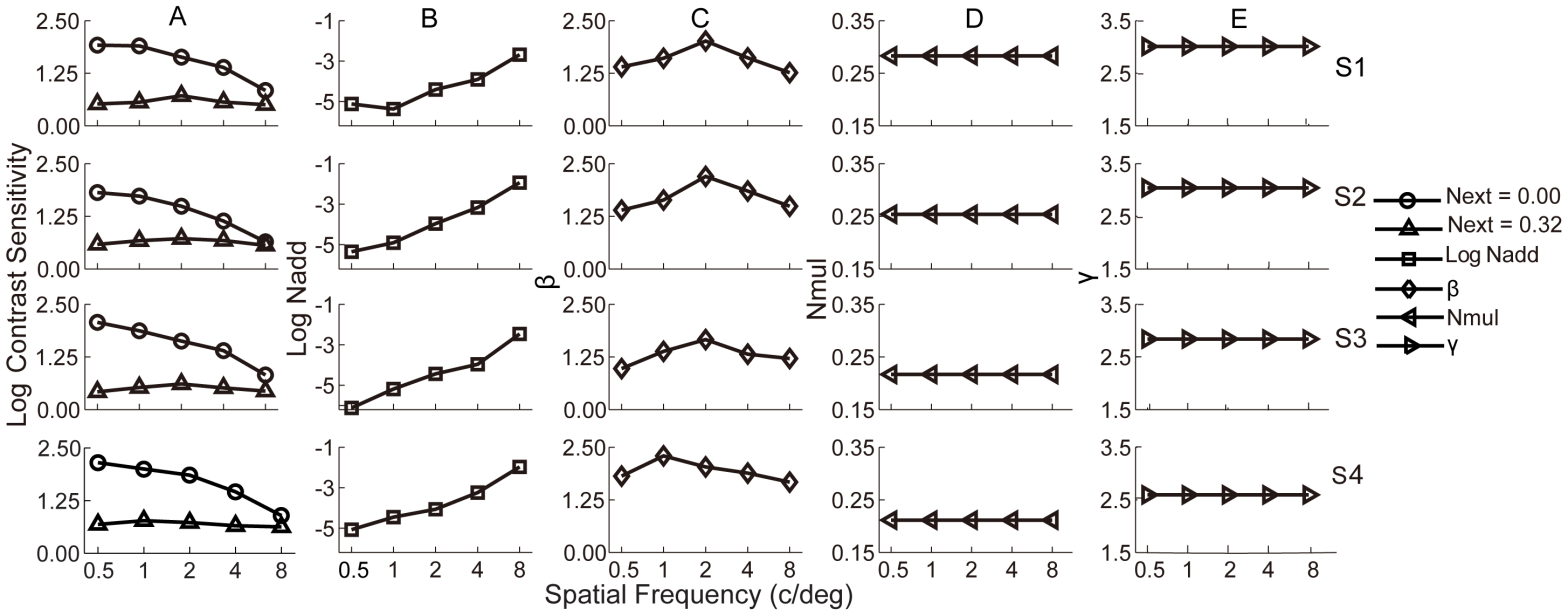
Results of perceptual template model analysis and contrast sensitivity functions. Column A: contrast sensitivity functions at zero (circle) and the highest external noise (triangle) levels are plotted as functions of spatial frequency; at zero external noise, the contrast sensitivity functions show a typical band-pass or low-pass profile. At high external noise, the contrast sensitivity functions have much lower amplitudes and don’t vary much with spatial frequency. Column B, C, D and E: *N_add_*, *β*, *N_mul_*, and *γ* as functions of spatial frequency, respectively.

**Figure 6:**
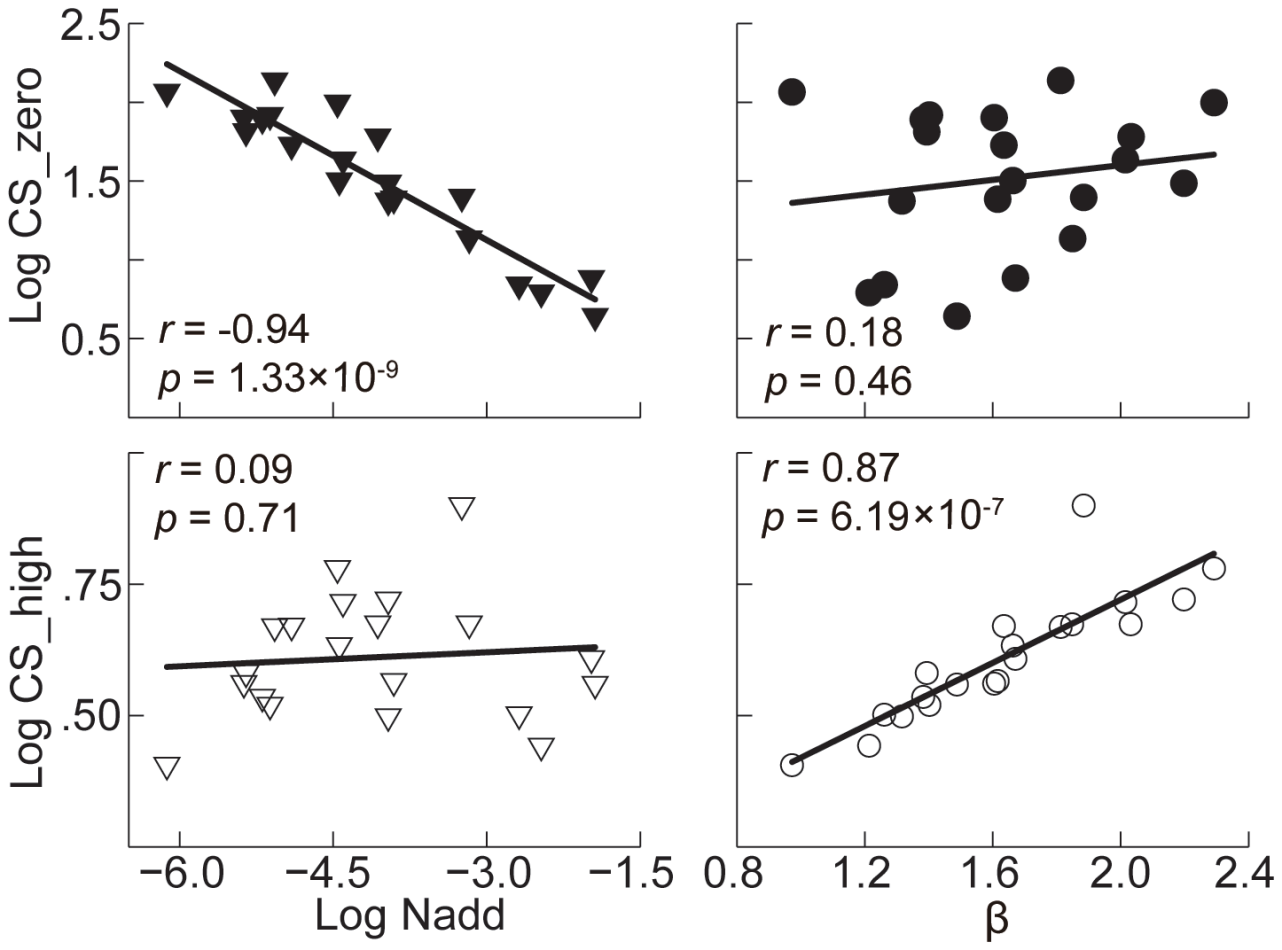
*N_add_* and *β* as functions of contrast sensitivity under zero and the highest noise conditions. The filled symbols represent the results of zero noise condition; the open symbols refer to the results of highest noise condition. Data from the four observers in the five spatial frequency conditions are pooled. CS_zero: contrast sensitivity in zero noise level (*N_ext_* = 0.00); CS_high: contrast sensitivity in highest noise level (*N_ext_* = 0.32).

Parameters of the best fitting PTM are listed in **Table 4** **.** In **Figure 5**, we plotted *N_add_*, *β*, *N_mul_*, and *γ,* together with the CSF at zero and the highest external noise levels, as functions of SF. It is interesting to note that internal additive noise changed more pronouncedly than the relative template gain. As SF increased from 0.5 to 8 c/deg, the internal additive noise increased by 2.43 to 3.66 log units (3.00±0.62, mean ± s.d.). As shown in **Figure 6**, CSF in the zero noise condition was almost solely correlated with internal additive noise (*r* = −0.94, *p* = 1.33×10^−9^), but not the relative template gain (*β*) (*r* = 0.18, *p* = 0.46). However, in the highest external noise condition, contrast sensitivity varied in a limited range across SFs, and was mainly correlated with the template gain (*β*) (*r* = 0.87, *p* = 6.19×10^−7^), but not the internal additive noise (*r* = 0.09, *p* = 0.71).

Following the suggestion of an anonymous reviewer, we also performed a comparison between the PTM and the LAM. The PTM reduces to the LAM when the multiplicative noise coefficient is 0 and the nonlinear transducer is 1. We compared the best fitting PTM in which only internal additive noise and template gain changed with spatial frequency with the LAM using a Chi-square test. The PTM provided significantly better fits to the data for all the subjects (all *p*<0.0000001).

## Discussion

By titrating the visual system with external noise and analyzing signal contrast threshold versus external noise functions with the perceptual template model, we demonstrated in the current study that the shape of CSF measured from 0.5 to 8 c/deg and in zero external noise is mainly a reflection of how internal additive noise changes with SF. Although template gain also changed significantly with spatial frequency, the magnitude of variation was very limited (less than a factor of 2). On the other hand, multiplicative noise and the nonlinear transducer remained constant with SF.

As the external noise increased from 0 to 0.32, the averaged contrast sensitivity decreased from 47.27 to 3.80, and the CSF became flatter (**Figure 4**). The finding that contrast sensitivity is largely independent of SF at high noise conditions is consistent with previous findings [39], [55], [56]. Our analysis suggested that the internal additive noise changed more profoundly with SF than the gain of the perceptual template did, consistent with the results found by Banks et al. [11], McAnany et al. [37], Rovamo et al. [39], and Tjan et al. [38] that CSF might be related with internal noise, and also consistent with Oruc et al. [40] and Kersten et al. [12] that external noise flattened CSFs. Our results suggest that model that employs CSF as the front-end SF filter to their inputs and a constant additive internal noise in all SF channels [33], [56], [57] might be incorrect and need to be revised. Although those models can explain the shape of the CSF in zero external noise, only the model presented in this paper can account for CSFs in a wide range of external noise conditions.

All the model parameters of the PTM are specified relative to the input signal and external noise stimuli presented to the subjects. We can further separate the contributions from the optics of the eye and subsequent stages of visual processing. Following the suggestion of an anonymous reviewer, we performed some additional analysis on our stimuli, taking into account of the modulation transfer function (MTF) of the eye. We filtered the signal and external noise images used in our experiment with a typical MTF for fovea detection [58] and a one-octave band-pass filter. The band-pass filter was used to model channel properties of the visual system [8]. We calculated the gain of the model to the signal grating and external noise in different center spatial frequency conditions. The MTF and the band-pass filter were:
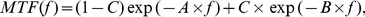
(7)




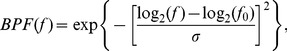
(8)where *f* is the spatial frequency in cycles per degree, *A*, *B*, and *C* are 0.172, 0.037, and 0.22, respectively [58]; *f*_0_ is the center spatial frequency, *σ* = 0.6 is the standard deviation of the band-pass filter with a half-height full-bandwidth of one octave.

The gain to the signal and external noise stimuli both decreased with central spatial frequency: the gains to signal stimuli with central frequencies of 0.5, 1, 2, 4, and 8 c/deg were 0.506, 0.469, 0.404, 0.308, and 0.193, respectively; the gains to the external noise were 0.139, 0.130, 0.114, 0.089, and 0.057, respectively; the ratios between the gains to signal and external noise were: 3.640, 3.608, 3.544, 3.461, and 3.386. Denoting *G_s_* and *G_n_* as the gain to the signal and external noise stimuli, we can incorporate the MTF into the PTM model:
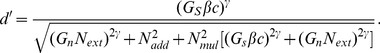
(9)


**Figure 7:**
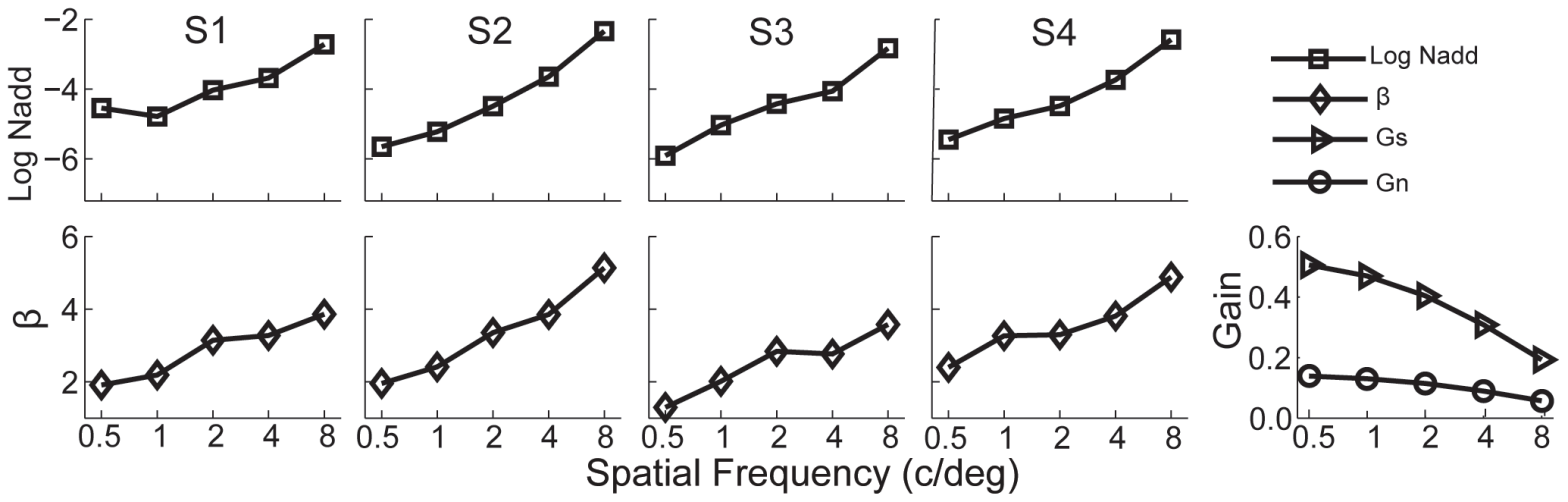
Modulation transfer function (MTF) and results of the best fitting MTF-incorporated PTM. The first four columns are *N_add_*, *β* as functions of SF for the four subjects, respectively, after taking the MTF of the eye into account. The gains of the eye to the signal and external noise stimuli in the five SF conditions are plotted in the last column.

We fitted the MTF-incorporated PTM to the data. As shown in **Figure 7**, model comparison revealed that the best model is still the one with both internal additive noise and template gain changing with spatial frequency but constant non-linear transducer and multiplicative noise coefficient. Averaged over four observers, internal additive noise increased by 2.77±0.66 log units (mean ± s.d.) and template gain increased by 136.4±38.9% as spatial frequency increased from 0.5 to 8 c/deg. The multiplicative noise coefficient and nonlinearity was constant across spatial frequencies. The contrast sensitivity function in the zero external noise condition was correlated with both internal additive noise (*r* = −0.94, *p* = 1.78×10^−9^) and template gain (*r* = −0.84, *p* = 4.24×10^−6^); in the highest external noise condition, neither the correlation between internal additive noise and CSF (*r* = −0.05, *p* = 0.82) nor the correlation between template gain and CSF (*r* = 0.29, *p* = 0.22) was significant. This additional analysis allowed us to separate contributions of the MTF of the eye and the rest of the visual system to the CSF.

In the current study, it took 18,000 trials to measure observer performance in 200 combinations of spatial frequency, external noise, and signal contrast using the method of constant stimuli (MCS). Although MCS provided full constraints of our model, the inefficient test procedure makes it impossible to apply our approach in clinical practice. Fortunately, we found that the slope of contrast psychometric functions was invariant across spatial frequencies and external noise levels. The finding is consistent with many previous observations [41], [43], [48], [54], [59] and supports the “homogeneity assumption” of slope for all pattern-detecting mechanisms [60]. The slope invariance provides an important regularity for us to exploit in developing quick adaptive methods to measure CSFs in different spatial frequency and external noise conditions [61].

Our approach may be applied to understand contrast sensitivity deficits in clinical populations. CSF deficits have been reported in amblyopia, dyslexia, glaucoma, and major depressive disorder. It will be very interesting to fully characterize the internal noise and spatial frequency gain profiles of the observers in those populations. Several studies have found that perceptual learning can significantly improve contrast sensitivity [22], [62]–[64]. It will also be interesting to obtain a snapshot of the visual system using the methods developed in this study before and after training to characterize the underlying mechanisms of the improvements.
